# Ambiguity in high definition: Gaze determines physical interpretation of ambiguous rotation even in the absence of a visual context

**DOI:** 10.3758/s13423-020-01776-x

**Published:** 2020-08-03

**Authors:** David Souto, Lily Smith, Jennifer Sudkamp, Marina Bloj

**Affiliations:** 1 Neuroscience, Psychology and Behaviour, University of Leicester, LE1 7HA, Lancaster Rd, Leicester, UK; 2 School of Optometry and Vision Sciences, University of Bradford, BD7 1DP, Richmond Road, Bradford, UK

**Keywords:** Visual perception, Eye gaze

## Abstract

Physical interactions between objects, or between an object and the ground, are amongst the most biologically relevant for live beings. Prior knowledge of Newtonian physics may play a role in disambiguating an object’s movement as well as foveation by increasing the spatial resolution of the visual input. Observers were shown a virtual 3D scene, representing an ambiguously rotating ball translating on the ground. The ball was perceived as rotating congruently with friction, but only when gaze was located at the point of contact. Inverting or even removing the visual context had little influence on congruent judgements compared with the effect of gaze. Counterintuitively, gaze at the point of contact determines the solution of perceptual ambiguity, but independently of visual context. We suggest this constitutes a frugal strategy, by which the brain infers dynamics locally when faced with a foveated input that is ambiguous.

The interaction of objects with ground surfaces provides valuable information for predicting and interpreting their motion (Gibson, 1950). Gaze movements allow the sampling of information about the object–ground relationship by foveating objects under scrutiny, thereby increasing the spatial resolution of the visual input. However, most of what we know about vision is about our perception of objects isolated from their physical environment. Consequently, we know little of the role of gaze in analyzing this contextual information.

Our rich natural visual world contains too much information to constantly and uniformly sample at a high resolution. This means that we often need to combine the visual input with prior assumptions about the physical world to disambiguate scenes. A well-known example of this is the light-from-above prior in our interpretation of shape-from-shading: By assuming that light comes from above, we can infer whether shadows are cast by a hollow or a bump (e.g., Adams, 2008). Prior assumptions of shape can drive color and brightness perception (Bloj & Hurlbert, 2002; Bloj, Kersten, & Hurlbert, 1999). More sophisticated inferences include generative models, used to predict the behavior of physical objects based on the everyday experience with Newtonian laws of motion (Ullman, Spelke, Battaglia, & Tenenbaum, 2017). Inferred motion trajectories (e.g., as affecting the ability to catch a baseball) have further been found to be influenced by the perception of gravity (McIntyre, Zago, Berthoz, & Lacquaniti, 2001; Monache, Lacquaniti, & Bosco, 2019).

Here, we focus on how an object’s movement can be disambiguated by combining a prior understanding of classical mechanics with sensory information. Frictional forces oppose the tendency of a moving ball to slide or skid, resulting in what is commonly defined as rolling without slipping. Rolling without slipping results in a combination of rotational and translational motion, where a ball would rotate clockwise during rightward translation and anticlockwise during leftward translation, as seen in Fig. 1a. Given the strong constraint friction puts on an object’s dynamics, an observer may solve ambiguity in a visual scene by inferring whether frictional forces are being exerted on a moving object. In a rolling ball scenario, foveating the potential point of contact with the ground surface may be considered as most informative regarding the presence or absence of friction. Yet prior work is inconclusive regarding the role of friction in disambiguating visual motion. When two random-dot spheres are rotated, the rotation direction can be made ambiguous in the absence of 3D cues indicating which dots are towards the front or the back of the sphere. Observers tended to perceive the spheres’ rotation in opposite directions when their surfaces touch and even when contact is only suggested behind a screen (Gilroy & Blake, 2004). This percept would be consistent with a physical interpretation of the scene being used to disambiguate visual information. However, recent investigations failed to replicate these findings and observed a well-known tendency for coupling instead (i.e., perceiving two ambiguous stimuli as moving in the same direction; Pastukhov & Zaus, 2018).

**Fig. 1:**
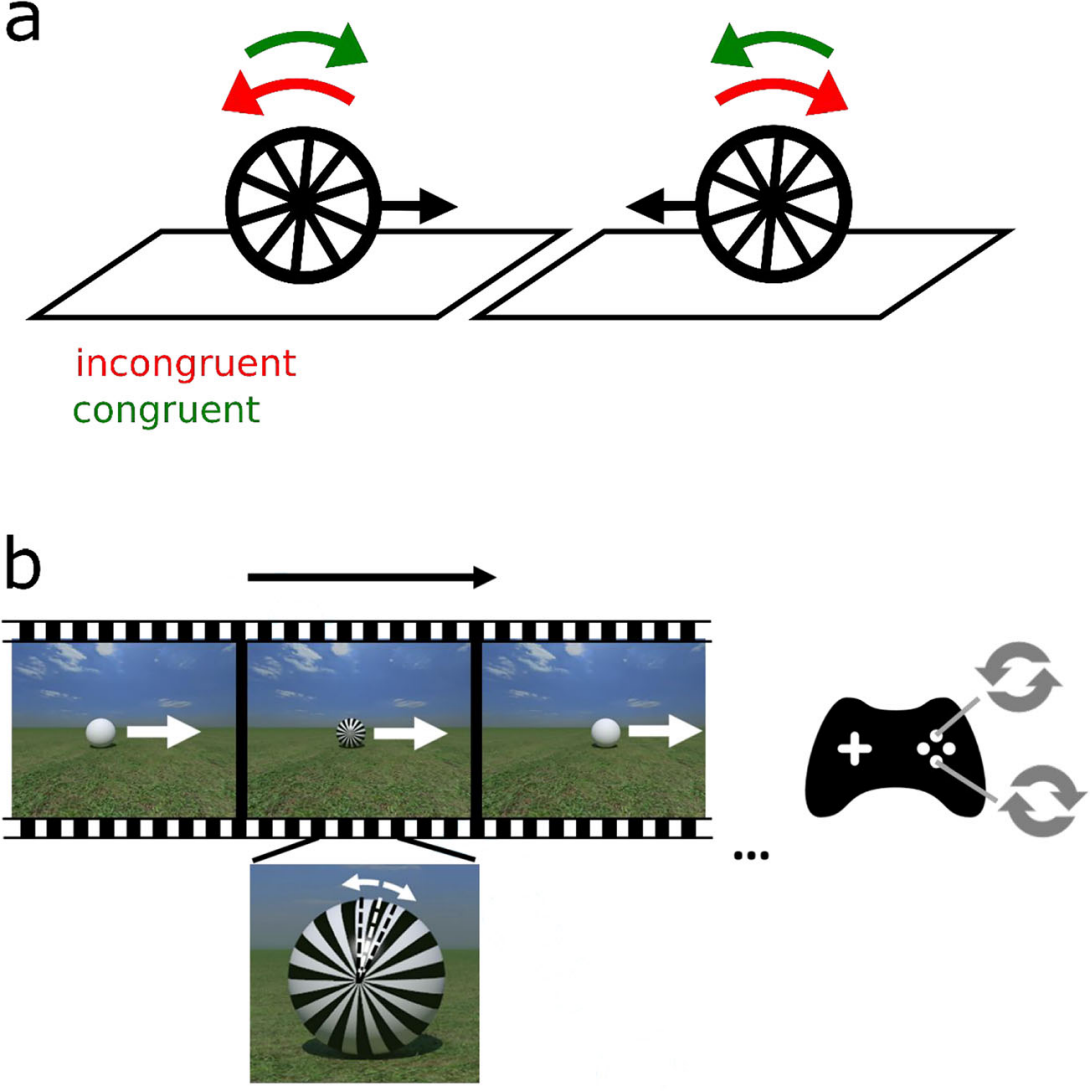
Experimental paradigm and perceptual interpretation depending on physical priors. **a** Illustration of what we call a percept that is congruent (green) or incongruent (red) with the direction of friction, assuming rotation without slipping. **b** Visual stimulation: A ball translates over the ground. For 800 ms, a radial pattern is displayed on the ball. The ball rotates by one spoke on every frame, such that the radial pattern rotation is perfectly ambiguous. Participants reported the clockwise or anticlockwise rotation of the radial pattern

In our study, we evaluated the effect of gaze and visual context on the physical interpretation of ambiguous motion by asking observers to judge the rotation of a virtual ball embedded or not in a naturalistic 3D scene. To uncover the influence of prior information, the kinematics of the ball was made informationally ambiguous by temporal subsampling, as shown in Fig. 1b and in video demonstrations (10.25392/leicester.data.11316557).

## Methods

### Participants

There were 19 participants in Experiment 1 (14 were female, 18–37 years old), including authors D.S. and L.S. There were 18 participants in Experiment 2 (13 female, 18–24 years old). An additional participant was excluded from the analysis in the absence of reliable eye-movement record. Most participants were undergraduate or postgraduate students from the University of Leicester’s School of Psychology. They were given either £6 or course credit in compensation for their time. Ethical approval was obtained from the School of Psychology, abiding by the guidelines of the Declaration of Helsinki.

Our choice of sample size was guided by prior studies, which have shown a large effect of physical interpretation on congruency in a pool of four participants (Gilroy & Blake, 2004). However, Experiment 1 was partly exploratory, as we had no specific predictions regarding the effect of gaze. We then chose the same sample size to confirm the effect of visual context and gaze in Experiment 2.

### Materials

Videos were displayed on a HP P1130 CRT screen, with a 1,280 × 1,024 pixels resolution at 85 Hz. Participants viewing distance was 61 cm. We tracked the right eye with a video-based eye tracker (EyeLink 1000, SR Research Ltd, Osgoode, Ontario, Canada). We used the Psychophysics Toolbox PB-3 on MATLAB to display videos that were generated by using custom scripts with the Persistence of Vision Ray Tracer (POV-Ray; Version 3.6; http://www.povray.org/).

### Visual stimulation and design

A still of the stimulus is shown in Fig. 1b; demonstrations can be seen in Movies 1–4, 10.25392/leicester.data.11316557). Movie 1 shows an ambiguously rotating ball that remains at the same location. This movie was not shown during either experiment, but it allows one to see the ambiguous rotation in a condition that is also perceptually ambiguous. Movie 2 shows a ball translating in the absence of a visual context (orthographic projection); Movies 3 and 4 show a ball translating on the grass under two contact conditions (contact or levitation). POV-Ray scripts were used to render 3D scenes at a 1,280 × 1,024 pixel resolution. Those scenes contained either a plain white or patterned ball. The rotation of a radial pattern projected onto the ball was made ambiguous by temporal subsampling. Images were compiled into subsampled 3-s video clips, showing rotation by 12° steps and the corresponding translation (see below). Clips’ resolution was 960 (width) × 768 (height) pixels, corresponding to 23 × 17 degrees of visual angle (deg). Every frame lasted for 160 ms. The white ball (diameter = 4 deg) translated across the screen from a location 3.6 deg leftward or rightward off the screen center at 2.6 deg/s. When the ball reached the screen center, it was covered in a radial pattern for five frames (for 800 ms). Ball rotation was ambiguous, as on every frame the pattern was rotated by one spoke (12°; see Fig. 1b). The radial frequency of the pattern was 15 Hz (15 dark and 15 white spokes).

### Ball rotation and translation

The pattern rotation on every frame was compatible with a ball rotation without slipping (i.e., the distance travelled by one point along the perimeter of the ball corresponded to the ball’s horizontal translation). We can obtain translation by *t* = *θ* ∙ *radius*, with *θ* representing rotation in radians meaning that the ball translated by 0.84 deg on every frame, or 2.6 deg per second.

In order to avoid any cue in the clips which could give away the true direction of rotation, we created two ambiguous (subsampled, with 12° steps) sequences, derived either from unambiguously clockwise or anticlockwise clip versions (1° steps), which we assigned randomly on every trial. A motion aftereffect (Anstis, Verstraten, & Mather, 1998) could have built up during long presentations, favouring rivalrous perception. We avoided rivalry by presenting patterns for a short period of time.

### Effect of contact cues (shadows) and gaze

We tested the effect of gaze position by cueing participants to pursue one of three spots on the ball at the beginning of each trial. The cue was a red dot (0.5 deg) flashed two times for 200 ms (on and off) at the beginning of the trial, when the ball was still static. The dot was located on the very top (+2 deg), center (0 deg), or very bottom (−2 deg) of the ball. We also tested the effect of shadows on congruent judgements, as the shadow beneath the ball either indicated contact with the ground (contact condition) or not (levitation condition), as shown in Movies 3 and 4. The levitation condition simulated a ball moving by a third of its size above the ground; by moving the camera up we ensured that the the ball had the same position within the image in both conditions. 

### Effect of visual context and gaze

In a second experiment, we tested congruence judgements when the background was upright, inverted or absent (no-context condition). In the latter, we wanted to remove any asymmetry in the stimulus (e.g., due to shading); therefore, we applied an orthographic projection and simulated a light source in front to the ball. In the inverted condition, the scene was flipped vertically around the image center.

### Procedure

Experiments took place in a dimly lit room. Participants’ head rested on a chin rest and front rest. Prior to the experiment, participants performed six training trials, in which the ball rotated unambiguously while translating leftward or rightward, and in which they received feedback on accuracy. Participants responded whether rotation was clockwise or anticlockwise by pressing a designated button located upward or downward on a game pad (see Fig. 1b). Arrows were drawn around the buttons, providing a constant reminder. A nine-point calibration of the eye tracker followed the training trials and was repeated if necessary.

In Experiment 1, each participant was shown a random sequence of 240 video clips, corresponding to a 3 (top, center, or bottom gaze position) × 2 (shadow indicates contact or levitation) × 40 (repetitions) factorial design. Direction was randomized (leftward or rightward translation). At the end of every clip, participants reported the clockwise or anticlockwise rotation of the radial pattern at their own pace.

In Experiment 2, each participant was shown a random sequence of 180 video clips: 3 (top, center, or bottom gaze position) × 3 (upright, inverted, no context) × 20 (repetitions). Direction was randomized (leftward or rightward translation). All variables were interleaved within a block, except visual context. The order of visual context blocks was balanced across participants. In this experiment, for the purpose of the analysis, gaze position was defined relative to the direction of gravity (i.e., what is “up” did not depend on the visual context).

### Data analysis

Responses were recoded as congruent relative to the direction of friction, as shown in Fig. 1a, when perceptual judgements on clockwise or anticlockwise rotation matched the translation of the ball (i.e., clockwise with rightward translation and anticlockwise with leftward translation). This meant that an individual bias to respond clockwise or anticlockwise will be cancelled out in congruent responses given a similar number of leftward and rightward trials.

To correlate gaze position with perceptual judgements, we averaged vertical gaze position over a 100-ms time window centered at 300 ms after the pattern onset. To select the most meaningful time window, we first ran a logistic regression predicting congruent responses as a function of vertical gaze over a window centered at different times, going from 0.5 to 3.5 s after movement onset per 50-ms steps. We did this for every individual in levitation and contact conditions, pooling data across eye-movement instruction. We then used the window for which we obtained the least total amount of deviance over all participants.

We used the lme4 (Bates, Maechler, Bolker, & Walker, 2015) package in R software environment to carry out statistical analyses (R Core Team, 2017). The effect of gaze and shadows on the proportion congruent responses were analyzed by using a logistic (logit link or *log*(*p*/(1 − *p*)) generalized linear mixed-effects model (Jaeger, 2008). “Participant” was specified as a random factor in the model; gaze and shadow type (coded as a dummy variable) as regressors; and *p*-values were derived from Wald’s *z* statistic. We confirmed that those were consistent with the *p*-values derived from a likelihood ratio test comparing the full model with a model without the effect in question (Jaeger, 2008). We used the Akaike information criterion (AIC) as a goodness-of-fit measure for model selection (Akaike, 1974). Experiments were not preregistered. Data and materials are available via the Open Science Framework (https://osf.io/sz8h9/).

## Results

Participants had to judge the rotation of ambiguously rotating patterns. We tested the effect of gaze and visual context on congruence judgements, as defined in Fig. 1a.

### Effect of shadows and gaze (Experiment 1)

**Fig. 2:**
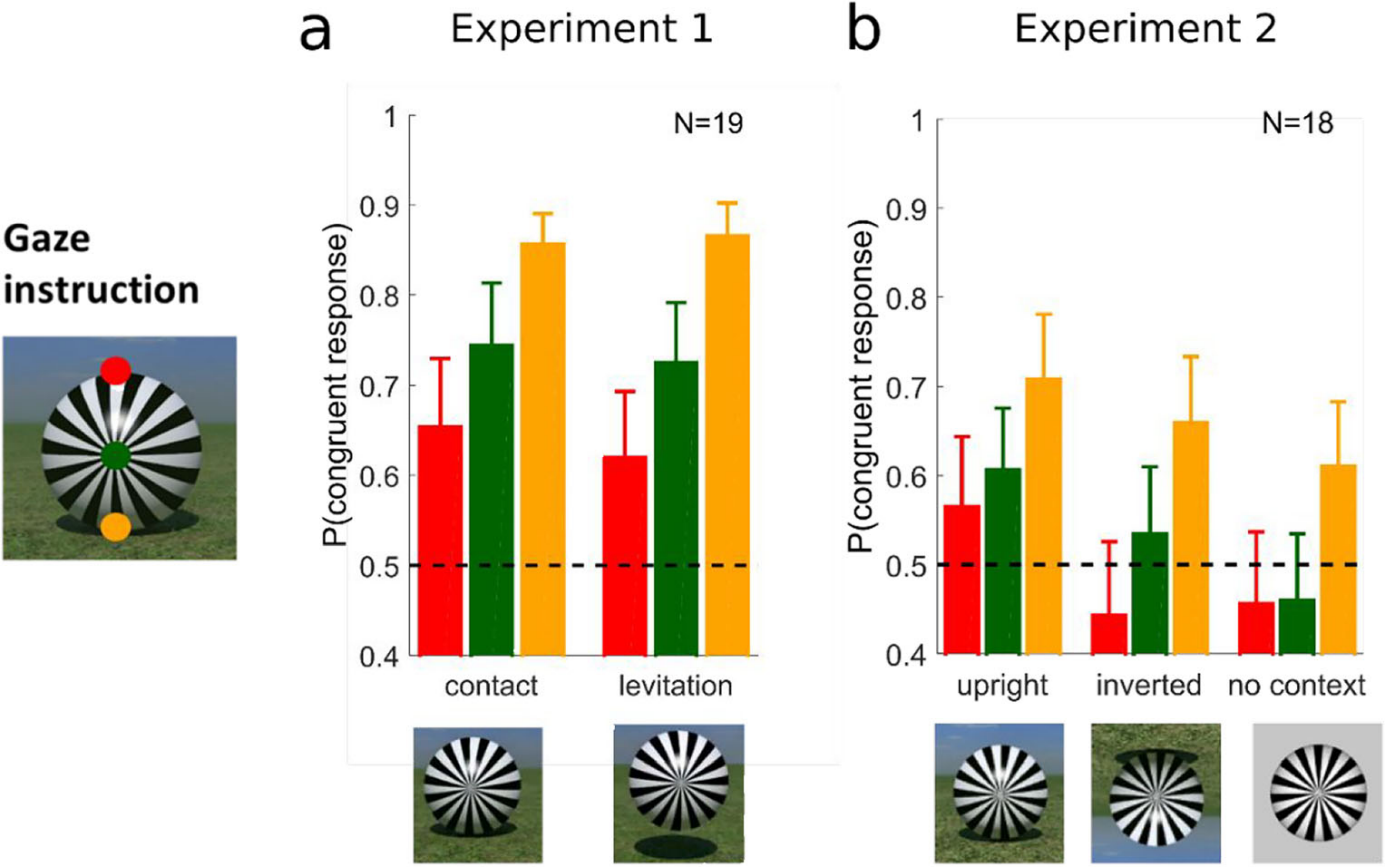
Proportion congruent judgements depending on instructions to look at different locations on the ball (red for the top, green for center, and orange for the bottom of the ball) and visual context in Experiments 1 (**a**) and 2 (**b**). Gaze position is always defined relative to the direction of gravity (i.e., as in the upright condition). Error bars represent the standard error of the mean

**Fig. 3:**
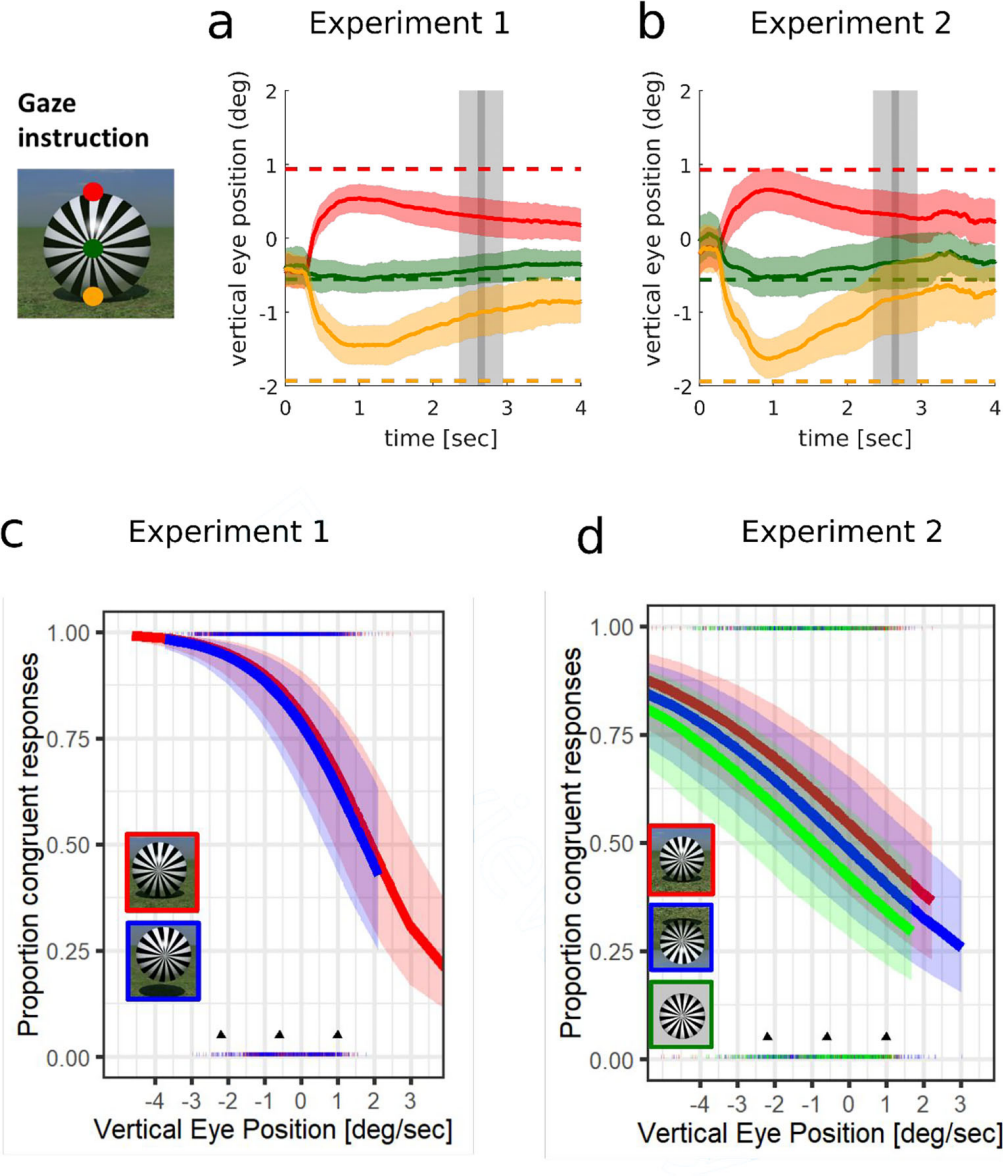
**a**–**b** Compliance with instructions to pursue different locations on the ball in Experiment 1 (**a**) and 2 (**b**). **c–d** Proportion congruent judgements depending on the actual gaze position (average for 250–350 ms after the presentation of the radial pattern; the light-grey vertical area indicates the averaging window) fitted by a logistic mixed-effects model, depending on shadow cues to contact (**c**, Experiment 1) and visual context (**d**, Experiment 2). The arrows heads near the *x*-axis indicate the different vertical locations on the ball according to gaze instructions. See the insets for the color code. **a–d** The colored shaded areas represent the 95% confidence interval

Overall, Fig. 2 shows that observers had a strong bias towards perceiving rotation as congruent with the direction of friction, with 74% (individually 31%–99%) congruent responses. The judgements did not depend on shadow cues (75% in the levitation condition, 74% in the contact condition), but they depended very strongly on gaze. Vertical gaze position ranged from −4 to 3 deg when the radial pattern was presented, with 0 corresponding to the center of the screen. As shown in Fig. 3a-b, observers were not very compliant with the instructions, since gaze veered towards the ball center during the presentation of the radial pattern, potentially reducing perceptual differences due to gaze. For this reason, we decided to analyze the effect of the actual gaze position on congruent responses during a critical time window (250-300 ms after presentation of the radial pattern; cf. Data analysis).

The best-fitting model is shown in Fig. 3c. This model indicated a highly significant effect of gaze, *SE* = 0.13, *z* = −6.146, *p* = 7.95 × 10^-10^, and no effect of shadow, *SE* = 0.084, *z* = −1.244, *p* = .213. A model that took only gaze into account had a lower AIC than the full model, and a chi-square test indicated that the log-likelihood of the reduced and full models were not significantly different, χ^2^(1) = 0.0837, *p* = .77. With the reduced model, the predicted proportion of congruent judgements was highest when looking at the bottom of the ball (*M* = 95% CI [89%, 98%]), still well above chance when looking at the middle, *M* = 85% (73%–93%), and hovering around chance level when looking at the top of the ball, *M* = 64% (44%–80%).

### Effect of visual context and gaze (Experiment 2)

Figure 2b and Fig. 3d show how vertical gaze instructions, actual gaze position and visual context relate to congruence judgements in Experiment 2, where observers saw different visual contexts in short blocks of trials (upright, inverted, and no-context conditions). The overall proportion of congruent responses was lower than in Experiment 1, but depending on the visual context (upright: 57%; inverted: 50%; no context: 50%), and had a wider range (individually 8% to 95%). However, a similar dependence with gaze was observed in all conditions, independently of the effect of visual context.

As in the first experiement, a logistic mixed-effects model was used to analyze the effect of gaze (averaged over the same temporal window) and visual context (coded as a dummy variable) on the proportion of congruent judgements. In the inverted condition, the whole scene was flipped vertically, which shifted the ball coordinates, since the upright-condition ball is not centered on the screen, but 0.6 deg below the center. We therefore shifted the gaze coordinates by the same amount for the inverted condition, so they aligned with the other conditions, before fitting the model.

The best-fitting model (Fig. 3d) showed that the proportion congruent responses dropped by about 10% without a context and 5% with an inverted context compared with the upright visual context. Gaze towards the point of contact increased the proportion congruent by about 25% compared with gaze on the top of the ball, to compare with a 31% increase in Experiment 1. The effect of gaze was statistically significant, *SE* = 0.803, *z* = −4.953, *p* = 7.31 × 10^-7^, as was the effect of visual context, *SE* = 0.0508, *z* = −4.196, *p* = 2.71 × 10^-5^. A reduced model, without an interactive term, had a lower AIC than the full model and had a similar likelihood, χ^2^(1) = 0.994, *p* = .318.

## Discussion

We postulated that gaze plays an important role in disambiguating dynamic information in relation to its physical context. We tested whether inferred friction with the ground determines the perception of an ambiguously rotating ball and how gaze helps to disambiguate rotation. We found that most often observers perceive the ball as rotating congruently with the direction of friction and that this effect depends much more on gaze than on visual context, with gaze located towards the point of contact (notional or visible) resulting in an increased percentage of congruent judgements.

How can we explain the effect of gaze on congruence judgements? When the ball is in contact with a visible ground, we can generate clear predictions based on a Bayesian framework (e.g., Weiss, Simoncelli, & Adelson, 2002). Congruence with a physical context could be thought of as driven by the influence of a prior (i.e., objects usually translate on a ground surface and are therefore subjected to frictional forces) optimally combined with sensory data. The information regarding rotation is equally ambiguous regardless of gaze position in our experiments, but the general reliability of visual information about frictional relationships is higher at the point of contact. We can then explain the effect of gaze by assuming that prior information regarding frictional relationships is only combined with sensory evidence when looking at the point of contact. If we only looked at the condition in which the ball rolls on the grass, the fact that foveation has such a role in disambiguation would strongly suggest that we rely on local visual cues to infer that the ball is in contact with the ground and subject to friction (e.g., Rolfs, Dambacher, & Cavanagh, 2013).

However, inverting or removing the visual context altogether had a much more modest effect on congruence judgements than gaze. Most remarkably, the presence or absence of a visual physical context did not interact with the effect of gaze. Observers continued to see the ball as moving congruently on most trials when looking at the point of contact, even when the top and bottom halves of the visual scene were identical. Therefore, what you see when you look at the top or bottom is not the determinant factor, but the fact you are looking at the *notional* point of contact. Indeed, whether the ball is pictured as levitating or in the absence of a visual context, the observer may still hold a strong prior assumption that rolling objects move on a ground surface, even if invisible (no-context condition) or even when there is some evidence that the object is not in contact with a ground plane (levitation). In particular, the straight-line translation of the ball may provide a cue that friction is being applied at the bottom, given the effect of gravity, which can override other visual cues (e.g. shadow cues).

In this case, a possible alternative explanation for the effect of gaze in specifying congruence relates to enforcing a coherent conscious experience of the foveated image that is consistent with the inferred physical context. Once gaze is already allocated to the point of contact and the relationship between surfaces (the ball and the ground) is still ambiguous, there is no sampling strategy that will disambiguate this stimulus further; the only remaining recourse is to rely on prior knowledge to resolve it. We suggest that this paradoxical effect constitutes a frugal heuristic, by which the brain disambiguates the foveal input based on prior knowledge, but spares the computational effort of generating predictions of physical dynamics at a global scale, given the fovea is also most often the focus of attention (Kowler, 2011).

There are different ways in which this heuristic could be implemented. The simplest one could be to favor congruent interpretations for stimuli lying in the upper visual field. This interpretation makes clear predictions. For instance, looking at the ball sideways (e.g., orienting the head 90 degrees relative to the vertical defined by gravity) would in that case change our impression of congruence. Further studies, in which viewing angle is dissociated from the direction of gravity, will be able to tell apart whether the physical interpretation in that situation is specified by visual familiarity (the ground is usually in the lower visual field) or the direction of gravity (Zago, McIntyre, Senot, & Lacquaniti, 2008).

Shadows provide another way of specifying the context, specifically contact with the ground (Madison, Thompson, Kersten, Shirley, & Smits, 2001). We also know that by changing the gap between an object and its projected shadow we can alter the perceived trajectory of a moving object (Kersten, Mamassian, & Knill, 1997). In our study, however, whether shadows indicated levitation or contact had no influence on the proportion of congruent judgements. This suggests that in our case contact is inferred based on the motion information, likely because rotation without slipping is a more likely interpretation given the visual information than levitation.

That is not to say that visual information had no sizable influence. Notably, there was a lower proportion of congruent responses in the second experiment compared to the first one. In the second experiment, observers experienced at times inverted and decontextualized environments before being presented with a normal context, which could have primed some observers to alternative interpretations of the scene (e.g., perceiving the sky as a surface on which the ball is rolling). Further studies would be needed to quantify how preexposure to different environments affect the type of physical interaction that is inferred, as this may tell us about how quickly we are able to update our priors.

Overall, the results pattern suggests an important role of gaze in disambiguating a percept. Gaze has been shown to have a role in specifying other object properties, such as determining object brightness, with fixation to brighter spots correlating with brighter object reflectance judgements (Toscani, Valsecchi, & Gegenfurtner, 2013a, 2013b). It would be of interest to investigate whether gaze has a similar effect across different kind of ambiguous (and rivalrous) stimuli. We may predict that the reliance on priors will depend on foveation. An analogous situation would be to expect shape-from-shading to reflect less the light-from-above prior in the periphery.

In conclusion, the visual system infers a physical context to disambiguate an object's motion, even when there is no visual context, but depending on gaze position. The fact that gaze correlates with solving ambiguity without adding visual information has wide implications in the study of visual function and the relation between vision and action. It could suggest not only that gaze serves movement (e.g., batting) by increasing the resolution of relevant information (e.g., the point of contact of a cricket ball), but that it may also gate inferential processes  (e.g. predicting how a ball will bounce).
